# Similar survival after endoscopic submucosal dissection and esophagectomy in early esophageal cancer and synchronous or metachronous head and neck cancer

**DOI:** 10.1186/s13019-024-02514-3

**Published:** 2024-02-04

**Authors:** Ruei-Ti Ke, Yu-Hsin Hsiao, Wei-Chen Tai, Shau-Hsuan Li, Chih-Chien Yao, Kai-Hao Chuang, Hsing-Hua Lai, Yu Chen, Li-Chun Chen, Hung-I Lu, Yen-Hao Chen, Chien-Ming Lo

**Affiliations:** 1 Department of Thoracic & Cardiovascular Surgery, Kaohsiung Chang Gung Memorial Hospital, 123 Ta-Pei Road, Niaosung Dist., Kaohsiung, Taiwan; 2 Department of Gastroenterology, Kaohsiung Chang Gung Memorial Hospital, Kaohsiung, Taiwan; 3 Department of Hematology-Oncology, Kaohsiung Chang Gung Memorial Hospital, Chang Gung University College of Medicine, Kaohsiung, Taiwan

**Keywords:** Early-stage esophageal cancer, Endoscopic submucosal dissection, Esophagectomy, Synchronous and metachronous head and neck cancer

## Abstract

**Background:**

Early-stage esophageal cancer is treated using endoscopic submucosal dissection and esophagectomy. Field cancerization in patients with early-stage esophageal cancer affects treatment outcomes and causes synchronous or metachronous head and neck cancers. We hypothesized that esophagectomy could provide better overall and relapse-free survivals in patients with esophageal cancer and synchronous or metachronous head and neck cancer.

**Methods:**

We retrospectively identified patients with early esophageal squamous cell carcinoma and synchronous or metachronous head and neck cancers. We separated the patients into endoscopic submucosal dissection and esophagectomy groups to compare overall and relapse-free survivals.

**Results:**

The study included 106 patients, 25 of whom underwent endoscopic submucosal dissection and 81 underwent esophagectomy. Overall and relapse-free survivals did not show significant differences between the two groups for both synchronous and metachronous head and neck cancers.

**Conclusions:**

Endoscopic submucosal dissection could provide similar overall and relapse-free survivals in patients with esophageal cancer and synchronous or metachronous head and neck cancer.

## Visual abstract

**Key question:**

Is esophagectomy better than endoscopic submucosal dissection in early esophageal cancer with head and neck cancer?

**Key findings:**

Results did not show significant differences between the two treatments.

**Take-home message:**

Endoscopic submucosal dissection could provide equal result in early esophageal cancer with head and neck cancer.

## Background

Esophageal cancer has poor outcomes; however, an early diagnosis with a curative treatment modality such as endoscopic submucosal dissection (ESD) or esophagectomy could provide better survival than a delayed diagnosis. In recent years, ESD has played an important role in the treatment of esophageal cancer. Unlike esophagectomy, ESD is less invasive and has fewer complications with the same oncologic effect.

Many studies have discussed and compared ESD and esophagectomy, and have shown similar or better results in terms of overall survival [1–5]. We referred these results and provided treatment for patients with early-stage esophageal cancer. However, synchronous and metachronous head and neck cancers have high incidence rates in Taiwan and worldwide [6, 7]. In such cases, ESD is difficult owing to use of combined procedures such as reconstruction surgery leading to anatomic changes and high-dose radiotherapy causing airway or neck stiffness. Field cancerization, which also alters treatment results, should be considered [8]. However, no studies discussed the result in early-stage esophageal cancer with head and neck cancer [1–5, 9, 10]. Since esophagectomy can remove most of the esophagus, we hypothesized that it would provide better overall and relapse-free survivals in such cases. We retrospectively reviewed patients from our esophageal cancer database.

## Materials and methods

### Patient population

Patients with esophageal cancer who underwent ESD or esophagectomy between November 2002 and December 2019 at the Kaohsiung Branch of Chang Gung Memorial Hospital were reviewed retrospectively. We evaluated patients’ pathological reports that showed the presence of stage 0 or I disease. All patients were assessed by a multidisciplinary team including surgeons, oncologists, radiation oncologists, radiologists, and gastroenterologists. Pretreatment evaluation included esophagogastroduodenoscopy (EGD), chest computed tomography with contrast enhancement, endoscopic ultrasonography, and positron emission tomography. The disease stage was confirmed using the 8th American Joint Committee on Cancer (AJCC) staging system. All patients will be listed in multiple multidisciplinary team meetings for treatment options. If the clinical survey shows that the lesion fits the criteria for endoscopic submucosal dissection [11], gastrointestinal physicians will discuss endoscopic submucosal dissection with the patients. If patients refuse or do not fit the criteria of endoscopic submucosal dissection, they will go on esophagectomy (since all of them are clinically operable by retrospective database selection). We excluded patients who underwent intervention at another hospital, were treated with radiofrequency ablation, had received adjuvant therapy such as concurrent chemoradiotherapy or chemotherapy alone before the intervention, and/or had unavailable medical records. Some patients were lost to follow-up for a long period. This study was approved by the Institutional Review Board of the Kaohsiung Chang Gung Memorial Hospital. The IRB certificate number is “202300639B0.”

### Thoracoscopic surgery and endoscopic submucosal dissection

Three different surgeons performed esophagectomies in these patients, and two experienced gastrointestinal endoscopists performed esophageal ESD. All patients underwent esophagectomy using the McKeown or Ivor-Lewis procedure. We used the same operating room settings, team members, and surgical devices for all patients. The esophagectomy specimens were sent to the pathology lab for reports including whole resected esophagus, thoracic and abdominal lymph nodes. Pathologists evaluated tumor and depth, lymphvascular invasion, resection margin and classified the stage in line with the American Joint Committee on Cancer eighth edition. About endoscopic submucosal dissection, the used equipment included flexible endoscopes (GIF-Q260J, Olympus, Tokyo, Japan) with a distal attachment (Olympus, Tokyo, Japan) and the Hybrid Knife™ water-jet system (ERBE, Tubingen, Germany) or Dual Knife-J™ electrosurgical knife (Olympus, Tokyo, Japan). The submucosal injection used during ESD included normal saline with bosmin and indigo-carmine with Hybrid knife™ and glycerol with bosmin and indigo-carmine with Dual Knife-J™ [12]. We performed a circumferential incision in the esophageal mucosa after marking the tumor margin with an electrosurgical knife, rather than dissecting the submucosa. In some cases with large tumors or severe submucosal fibrosis, we used the traction method with an endoscopic clip and dental trephine to complete the submucosal dissection. Hybrid-ESD delimited as resection was completed using Captivator II™ Single Use Snare (Boston Scientific, Natick, MA, USA) [12] after circumferential incision and adequate partial submucosal dissection. An electrosurgical knife or the Coagrasper™ Hemostatic Forceps (Olympus, Tokyo, Japan) was used for hemostasis during and after complete ESD to prevent post-ESD bleeding [12]. Sucralfate gel was sprayed directly on the post-ESD wound to assess the presence of a possible minor bleeder. After ESD, the specimens were oriented by fixing the periphery with thin needles on a plate immediately before immersion in formalin. Post-ESD specimens were sent for pathological examination and serially sectioned at 2 mm intervals. Both the lateral and vertical margins were assessed microscopically, and the depth of tumor invasion was evaluated based on the degree of differentiation and lymphovascular infiltration. R0 resection was defined as en bloc resection with all histologically negative margins.

### Overall survival and relapse-free survival

We used two scales to compare the results of ESD and esophagectomy. Overall survival was defined as the period from the first intervention date of esophagectomy or ESD to the last contact date. If a patient died (regardless of the cause of death), we delimited it as an event. If a patient survived, it was delimited and censored. Relapse-free survival was defined as the period from the first intervention date of esophagectomy or ESD to the last contact date. If a patient died or had a computer tomography scan or EGD showing progression in the regional lymph node or residual esophagus, we delimited it as an event. If an image was stable, it was delimited and censored.

### Synchronous and metachronous head and neck cancer

Synchronous tumors were confirmed within 6 months of the identification of the index tumor, and metachronous tumors were confirmed more than 6 months after the index tumor [13].

### Statistical analysis

Data were analyzed using MedCalc® Statistical Software version 20 (MedCalc Software Ltd, Ostend, Belgium; https://www.medcalc.org; 2021). A χ2 test was used to contrast data between the two groups. The Kaplan–Meier method was used for univariate survival analysis, and the difference between survival rates was analyzed using a log-rank test. Factors were entered into a Cox regression model in a forward manner to analyze their relative prognostic importance. For all analyses, two-sided tests of significance were used, with *p* < 0.05 indicating statistical significance.

## Results

### Patient characteristics and esophagectomy result

In total, 106 patients who underwent ESD and esophagectomy were included in this study. The mean age was 55.36, median age was 55, and age range of the patients was 40–80 years. A total of 51.9% of patients had comorbidities in the survey before the procedures. Two patients were diagnosed with AJCC 8th edition clinical stage III tumor owing to N2 disease. Further, 19.8%, 50%, and 30.2% of the primary tumor locations were in the upper, middle, and lower thirds of the esophagus, respectively. Grade 2 (moderately differentiated) tumors comprised a high proportion of pathological tumor grades (73.6%). Additionally, 50% of patients showed synchronous head and neck cancer and 22.6% of patients showed metachronous. Clinical features of the 106 patients are shown in Table 1.

**Table 1:** Clinicopathologic factors of 106 patients with 8th AJCC pathologic stage 0 or I esophageal squamous cell carcinoma

Factors	No. of patients (Percentage)
Age (years) (range: 40–80, mean: 55.36, median: 55)	
Current smoker	
Yes	23 (21.7%)
No	83 (78.3%)
Comorbidity (diabetes mellitus, liver cirrhosis, hypertension, esophageal reflux disease, CAD)	
0	51 (48.1%)
1	55 (51.9%)
Clinical 8th AJCC stage	
0	10 (9.4%)
I	80 (75.5%)
II	14 (13.2%)
III	2 (1.9%)
Clinical T stage	
0 (Tis)	11 (10.4%)
T1	79 (74.5%)
T2	16 a(15.1%)
Clinical N stage	
N0	97 (91.5%)
N1	7 (6.6%)
N2	2 (1.9%)
Primary tumor location	
Upper	21 (19.8%)
Middle	53 (50.0%)
Lower	32 (30.2%)
Pathological tumor grade	
0 (Tis)	13 (12.3%)
1	8 (7.5%)
2	78 (73.6%)
3	7 (6.6%)
Synchronous head and neck cancer	
No	53 (50.0%)
Yes	53 (50.0%)
Metachronous head and neck cancer	
No	87 (82.1%)
Yes	19 (17.9%)

AJCC, American Joint Committee on Cancer

Eighty-one patients received thoracoscopic esophagectomy using the McKeown or Ivor-Lewis maneuver. The average age of the 81 patients was 54.31 and median age was 54 (range, 40–75) years. Fifty-eight patients did not have complications after esophagectomy, and 92.6% had histologically negative margins. The characteristics of the 81 patients who underwent esophagectomy are listed in Table 2.

**Table 2:** Surgical factor and complications in 81 patients with 8th American Joint Committee on Cancer pathologic stage 0 or I esophageal squamous cell carcinoma

Factors	No. of patients (Percentage)
Age (years) (range: 40–75, mean: 54.31, median: 54)	
Blood transfusion during operation	
Yes	10 (12.3%)
No	71 (87.7%)
Operative time	
< 8 h	17 (21.0%)
≥ 8 h	64 (79.0%)
Post-operative intensive care unit stay	
≤ 3 days	13 (16.0%)
> 3 days	68 (84.0%)
Hospital stay	
≤ 20 days	30 (37.0%)
> 20 days	51 (63.0%)
Complication	
None	58 (71.6%)
Pulmonary	15 (18.5%)
Conduit	6 (7.4%)
Vocal cord palsy	2 (2.5%)
Tumor grade	
0 (Tis)	4 (4.9%)
Grade 1	7 (8.6%)
Grade 2	63 (77.8%)
Grade 3	7 (8.6%)
Perineural invasion	
No	80 (100%)
Lymphovascular invasion	
Yes	5 (6.2%)
No	76 (93.8%)
Extracapsular invasion	
No	80 (100%)
R0 resection	
Yes	75 (92.6%)
No	6 (7.4%)

### Esophagectomy and endoscopic submucosal dissection

We attempted to compare ESD and esophagectomy results. We found some parameters that showed obvious differences between the ESD and esophagectomy groups, such as age, clinical stage, clinical T stage, tumor grade, and pathological stage. We attempted to perform subgroup analysis between pathological T stages 0 (Tis) and T1a. After excluding pathological stage T1b lesions, differences between the two groups were eliminated. We excluded patients whose pathologic specimen showed T1b lesions because all these patients received diagnostic endoscopic submucosal dissection rather than treatment. All of them went on esophagectomy or concurrent chemoradiotherapy if surgery was not feasible. The interventional features of ESD and esophagectomy are listed in Table 3.

**Table 3:** Intervention features of patients with 8th AJCC pathologic stage 0 and I esophageal squamous cell carcinoma

Parameters			Management		
			Endoscopic submucosal dissection (25)	Esophagectomy (81)	p-value
Age (years) (Mean ± standard deviation)			58.8 ± 9.4	54.3 ± 8.0	0.021*
Clinical 8th AJCC stage	0		7	3	0.0007*
	I		18	62	
	II		0	14	
	III		0	2	
Clinical T stage	Tis		7	4	0.0006*
	T1		18	61	
	T2		0	16	
Clinical N stage	N0		24	73	0.60
	N1		1	6	
	N2		0	2	
Tumor grade	0 (Tis)		9	4	0.0003*
	1		1	7	
	2		15	63	
	3		0	7	
Primary tumor location	Upper		6	15	0.11
	Middle		8	45	
	Lower		11	21	
Pathologic 8th AJCC stage	0		11	6	0.0001*
	IA		0	3	
	IB		14	72	
R0 resection	Yes		24	75	0.55
	No		1	6	
Synchronous H&N cancer (clinical stage) (Total: 53)	I		3	9	0.56
	II		1	7	
	III		0	4	
	IV		8	21	
Metachronous H&N cancer (clinical stage) (Total: 19)	I		0	4	0.51
	II		1	3	
	III		0	0	
	IV		3	8	
Management of H&N cancer	Operation		4	18	0.18
	CCRT		6	25	
	CCRT + OP		3	3	
	RTO		1	0	

*: Statistically significant. х^2^ test or t test was used for statistical analysis

AJCC, American Joint Committee on Cancer; H&N, head and neck

### Overall survival and relapse-free survival

Several factors matched between overall and relapse-free survivals. Clinical and T stages had significant impact on overall and relapse-free survivals. Age and tumor grade affected the relapse-free survival. Primary tumor location affected overall survival, but not relapse-free survival. Clinical N stage, pathologic stage, pathologic T stage, R0 resection, procedure, synchronous head and neck cancer, and metachronous head and neck cancer did not affect overall and relapse-free survivals. All these parameters are outlined in Table 4.

**Table 4:** Results of univariate analysis for overall and relapse-free survivals in 106 patients with 8th AJCC pathologic stage I esophageal squamous cell carcinoma

Factors	No. of patients	OS		RFS	
		3-year OS (%)	p	3-year RFS (%)	p
Age					
< 55 years	55	77%	0.075	74%	0.0291*
≥ 55 years	51	74%		64%	
Clinical 8th AJCC stage					
0	10	100%	0.0028*	90%	0.0062*
I	80	75%		70%	
II	14	50%		43%	
III	2	100%		100%	
Clinical T stage					
Tis	11	100%	0.0055*	91%	0.0125*
T1a	79	76%		70%	
T1b	16	56%		50%	
Clinical N stage					
N0	97	75%	0.47	70%	0.49
N1	7	71%		57%	
N2	2	100%		100%	
Tumor grade					
Grade 0 (Tis)	13	92%	0.486	92%	0.05*
Grade 1	8	63%		63%	
Grade 2	78	75%		66%	
Grade 3	7	86%		86%	
Primary tumor location					
Upper	21	76%	0.0343*	71%	0.075
Middle	53	65%		60%	
Lower	32	91%		84%	
Pathologic 8th AJCC stage					
0 (Tis)	17	94%	0.198	94%	0.249
IA	3	67%		67%	
IB	86	72%		65%	
Pathologic 8th AJCC T stage					
0	17	94%	0.193	94	0.25
T1a	34	78%		69	
T1b	55	68%		63	
R0 resection					
Yes	99	75%	0.438	69%	0.754*
No	7	83%		86%	
Procedure					
Endoscopic submucosal dissection	25	87%	0.118	83%	0.085
Esophagectomy	81	72%		66%	
Synchronous H&N cancer					
No	53	82%	0.593	77%	0.494
Yes	53	69%		63%	
Metachronous H&N cancer					
No	87	73%	0.471	68%	0.605
Yes	19	79%		74%	

*Statistically significant

OS, overall survival; RFS, relapse-free survival; AJCC, Americal Joint Committee on Cancer; H&N, head and neck

### Synchronous and metachronous head and neck cancer

**Fig. 1:**
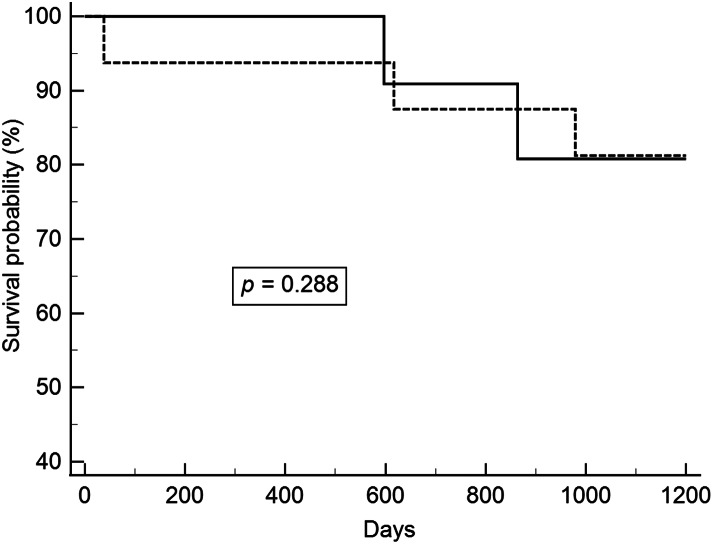
Overall survival between ESD and esophagectomy in pathologic stages 0 and T1a esophageal cancer and synchronous head and neck cancer (Solid line: ESD; Dot line: Esophagectomy). ESD, endoscopic submucosal dissection

**Fig. 2:**
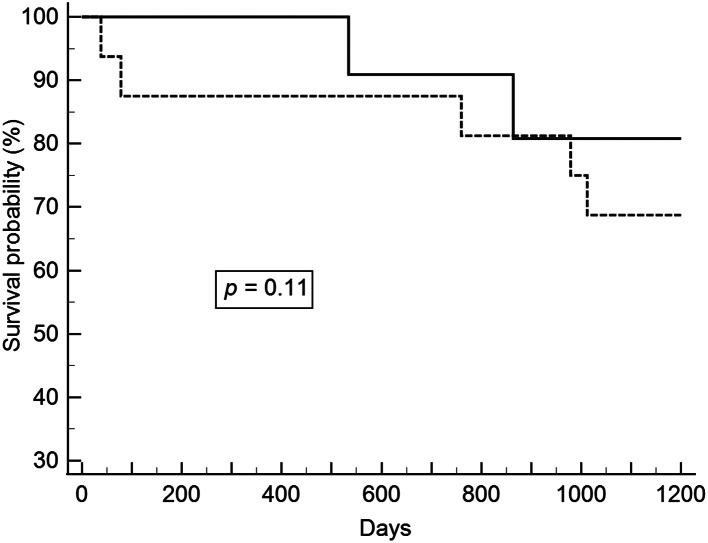
Relapse-free survival between ESD and esophagectomy in pathologic stages 0 and T1a esophageal cancer and synchronous head and neck cancer (Solid line: ESD; Dot line: Esophagectomy). ESD, endoscopic submucosal dissection

**Fig. 3:**
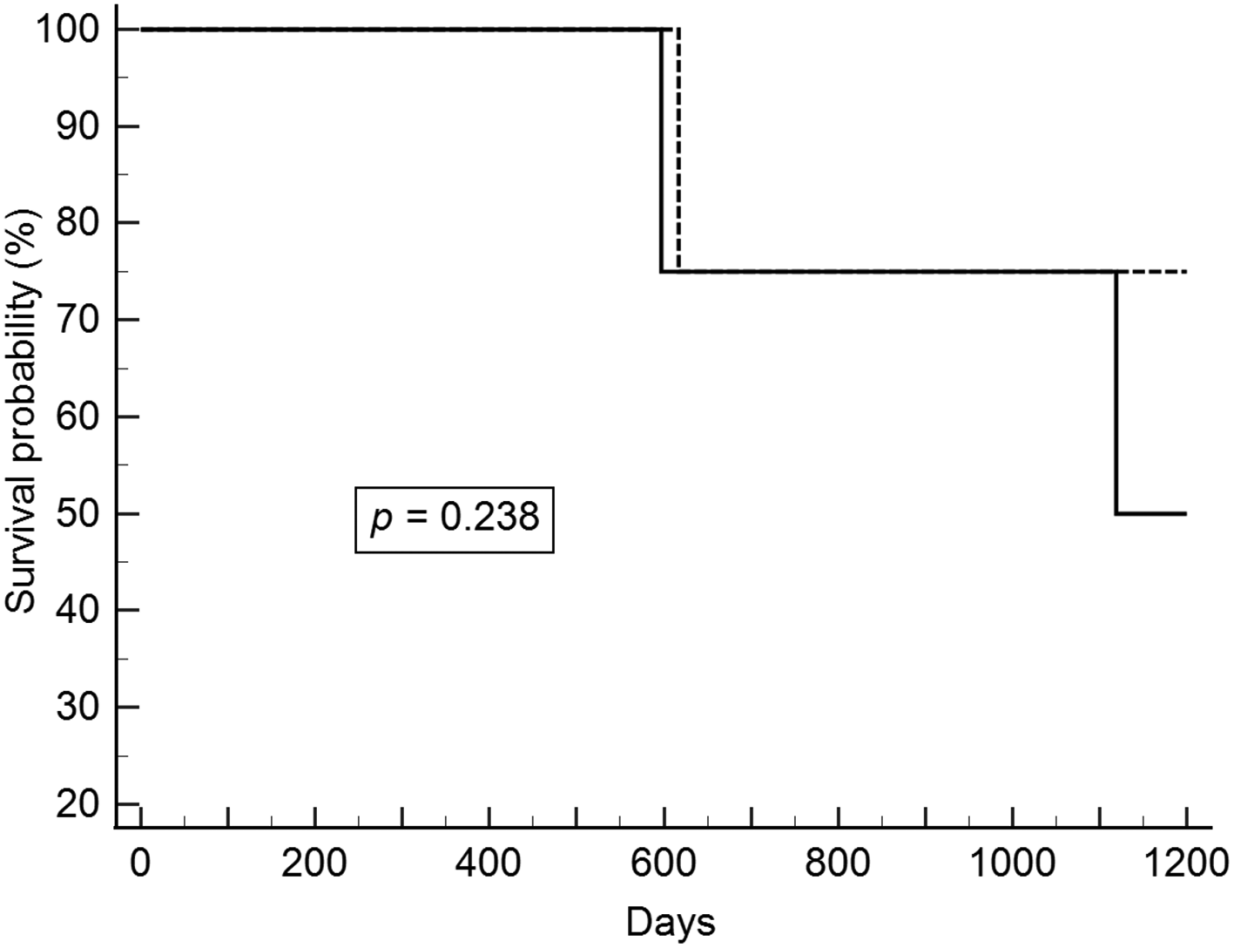
Overall survival between ESD and esophagectomy in pathologic stages 0 and T1a esophageal cancer and metachronous head and neck cancer (Solid line: ESD; Dot line: Esophagectomy). ESD, endoscopic submucosal dissection

**Fig. 4:**
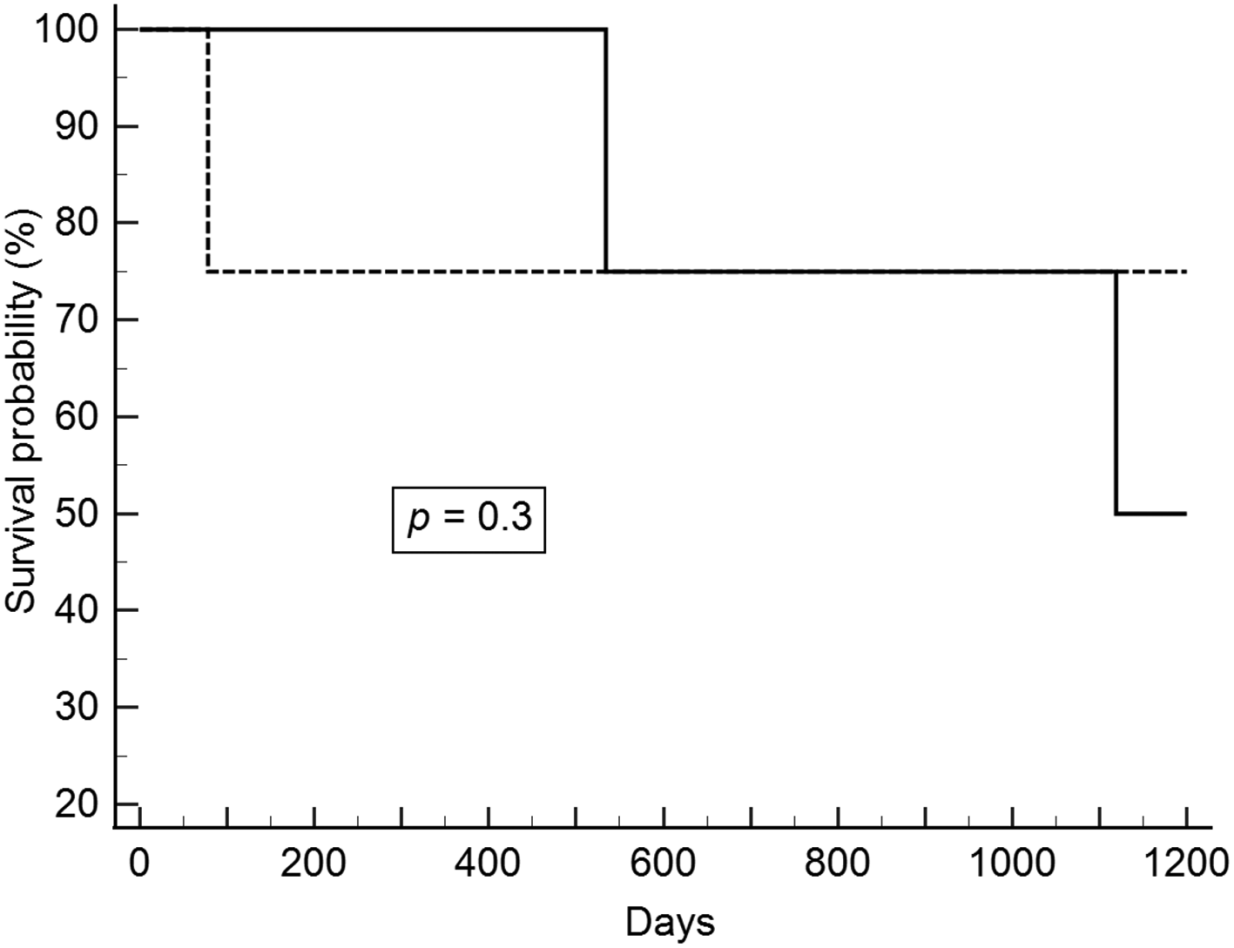
Relapse-free survival between ESD and esophagectomy in pathologic stages 0 and T1a esophageal cancer and metachronous head and neck cancer (Solid line: ESD; Dot line: Esophagectomy). ESD, endoscopic submucosal dissection

After excluding patients with pathological stage T1b lesions, we analyzed patients with synchronous and metachronous head and neck cancers. ESD and esophagectomy did not have a significant effect on overall and recurrent-free survivals. The survival curves are shown in Figs. 1, 2, 3 and 4.

## Discussion

In this study, we analyzed the outcomes of ESD and esophagectomy in patients with early-stage esophageal cancer and synchronous or metachronous head and neck cancer. ESD did not show significantly inferior results to esophagectomy in terms of overall and relapse-free survivals. This result supports the hypothesis that esophagectomy leads to better overall or relapse-free survival in patients with esophageal cancer and synchronous or metachronous head and neck cancer.

Field cancerization is a hypothesis of synchronous and metachronous head and neck cancers in patients with esophageal cancer leading to poor survival [14]. This concept has been proven in several publications, not only in cell models, but also in molecular pathway theory [15–17]. Some literatures reported contrasting results, indicating that synchronous or metachronous cancer did not affect survival outcomes [18]. Esophagectomy resects the entire esophagus and reduces the volume of field cancerization. We hypothesized that esophagectomy could provide better overall and relapse-free survivals in patients with early-stage esophageal cancer and synchronous or metachronous head and neck cancer. However, the results of the present study did not support this hypothesis. There are several reasons for this. First, complications after esophagectomy can reduce overall and relapse-free survivals. As shown in Table 2, the surgical complication rate could reach up to 20%. ESD did not result in major complications (not only pulmonary complications but also conduit or other complications), which led to better overall and relapse-free survivals. Another reason for this result is the small number of patients; 30 patients were enrolled to compare overall and relapse-free survivals after excluding patients with pathologic stage T1b lesion. Many studies have shown similar results with ESD and esophagectomy for early-stage esophageal cancer [1–5]. Most of these studies focused on the overall survival between ESD and esophagectomy using different statistical methods, including propensity score matching, prospective clinical trials, and multicenter clinical trials. None of them mentioned that synchronous or metachronous head and neck cancer would have an impact on overall or relapse-free survival.

Synchronous or metachronous head and neck cancer in patients with esophageal cancer could cause difficultly in performing ESD because of the use of a surgical intervention (reconstruction and bizarre anatomic changes) or concurrent chemoradiotherapy (rigidity and fibrosis of neck muscle and mouth opening limitation). Esophagectomy is a treatment option for these patients; however, few studies have compared the results of the two procedures in this group of patients. Our study illustrates that ESD provides similar results (even better in trend) in overall and relapse-free survivals in patients with pathologic stage T1a or Tis disease.

The reason for a high relapse rate even in the pathological Tis group is field cancerization. In our study, three patients suffering from recurrences were in the pathological Tis group, and two of them had synchronous or metachronous head and neck cancer. Another reason for the high relapse rate is small study groups. Only 17 patients with pathological Tis were found in our database.

The limitations of this study are its retrospective design and small number of cases. If more patients are enrolled, the trend of the benefits of ESD will be more obvious. A retrospective review study will conceal more confounding factors such as clinical diagnosis accuracy. Clinical T and N stage difference showed some impact in different groups as shown in Tables 3 and 4. We tried to eliminate it by excluding patients with pathologic T1b lesions. Another limitation is that additional subgroup analysis, such as head and neck cancer stage, may be a confounding factor that we could not determine because of the small number of cases.

## Conclusions

In conclusion, ESD provides results like esophagectomy in patients with esophageal cancer and synchronous and metachronous head and neck cancers. Multicenter or large database studies in the future may provide more robust evidence for the results of these procedures.

## Data Availability

All data generated or analyzed during this study are included in this published article.

## Abbreviations

ESD: endoscopic submucosal dissection
EGD: esophagogastroduodenoscopy
AJCC: American Joint Committee on Cancer
